# Comparison of a Smartphone App to Manual Knee Range of Motion Measurements

**DOI:** 10.1016/j.artd.2021.09.005

**Published:** 2022-04-04

**Authors:** Paul W. Knapp, Robert A. Keller, Kathryn A. Mabee, Jing Shi, Raji Pillai, Nicholas B. Frisch

**Affiliations:** a Ascension Macomb-Oakland Hospital, Madison Heights, MI, USA; b Orthopaedic Surgery, Ascension Providence Rochester Hospital, Rochester, MI, USA; c Medical Affairs Consulting, Inc., San Francisco, CA, USA

**Keywords:** Range of motion, Musculoskeletal pathology, Telerehabilitation, Remote monitoring, Digital technology, Musculoskeletal recovery platform

## Abstract

**Background:**

Digital technology has emerged as a useful tool for preoperative and postoperative patient engagement and for remote patient monitoring. Smartphones are equipped with motion-sensing technology, and apps can be designed which use these features to create a simple method for measuring range of motion. The purpose of this study was to determine the accuracy of digital technology in assessing knee range of motion using a smartphone app, compared to traditional goniometric measurements in an office setting.

**Methods:**

Fifty-three (53) patients in a clinical practice were enrolled between October 2019 and March 2020. Three separate measurements were taken during the patient encounter: (1) the surgeon, (2) the app, and (3) the physical therapist. Intraclass correlations were computed to assess the agreement between (1) the surgeon and app and (2) that between the physical therapist and surgeon.

**Results:**

When measuring flexion, the correlation between either the surgeon or therapist with the app was good, whereas the comparison between the surgeon and therapist was moderate. All extension measurement comparisons, between the app, surgeon, and therapist, showed moderate correlation. Limits of agreements showed that 80% of the difference between surgeon and app is within 10 degrees for extension and 11 degrees for flexion. Body mass index did not affect the accuracy of the measurements.

**Conclusion:**

Digital app measurements were comparable to measurements made by either a surgeon or physical therapist with a manual goniometer in the clinical setting and may be beneficial for measuring and monitoring patients’ range of motion remotely.

## Introduction

Increasing patient engagement has been suggested to improve surgical outcomes and patient satisfaction after orthopedic surgical procedures [[1], [2], [3]]. It has been shown that more preoperative patient activation, or a patient’s willingness to engage in adaptive health behaviors, is related to better overall pain scores and greater satisfaction after total joint arthroplasty [4]. In addition, the conventional method of postoperative outcome monitoring with frequent office visits may be inconvenient and costly for patients after surgery. Furthermore, as recent events have unfolded with the COVID-19 pandemic, there has been a significant shift toward remote patient contact. Digital technology has emerged as a useful tool to not only engage patients in the preoperative and postoperative period but is also used as a mechanism for patient monitoring remotely. Implementation of digital technology for remote monitoring has the potential to not only improve outcomes but also potentially reduce costs and readmissions [5].

A critical aspect of providing care in patients with acute or chronic knee pathology is assessing range of motion (ROM). This is typically performed in the office setting by the provider, or alternatively, by physical therapy. Traditionally, these measurements are made with the use of a goniometer. Advances in digital technology provide an alternative form of measuring ROM using various digital devices or smartphone applications. Smartphones may be equipped with accelerometers, magnetometers, and goniometers, or alternatively, apps have been designed with algorithms to use some or all these features to create a simple method of measuring ROM in the extremities.

While goniometry, the established method for ROM measurements, has been reported to be accurate [6,7], there has been an increasing interest in the exploration of using newer digital technology in assessing ROM in the knee [8], wrist [9], and elbow [10]. Although there has been some promise for the accuracy of cell phone goniometers recently, more data are needed to validate these tools for clinical use [11]. The purpose of this study is to determine the correlation of manual goniometric and digital application measurements in assessing knee ROM using a smartphone app compared to traditional goniometric measurements in an office setting.

## Material and methods

Institutional review board approval was obtained through our institution. Fifty-three patients in a clinical practice between October 2019 and March 2020 were enrolled. Patients were included regardless of the reason for clinical visit; they did not need to be surgical patients as the goal of the study was to determine the correlation between manual goniometric and digital measurements. Patients were excluded if there was a history of acute traumatic injury to the knee limiting ROM. In addition to ROM, patient height, weight, and body mass index (BMI) were also recorded. BMI measurements were included to address clinical concerns with accuracy associated with patients’ body habitus.

### Range of motion measurements

Three separate measurements were taken during the patient encounter: (1) the surgeon, (2) the app, and (3) the physical therapist (PT). The investigating surgeon took initial measurements during the office visit with a manual goniometer using a standard technique. Bilateral knee measurements were taken on all patients in full extension and flexion. Patients were asked to maximally extend and flex the knee to assess active ROM. Passive ROM was not obtained. The greater trochanter, center of rotation of the knee, and the lateral malleolus were used as landmarks for measurement in full extension and maximal flexion. A senior orthopedic resident supervised as patients obtained ROM measurements using a smartphone app (hereafter referred to as “App”) designed for remote ROM monitoring (PeerWell, San Francisco, CA). The App vocalizes instructions to the patient or provider on the proper technique. The protocol involves placing the phone on the anterior thigh and, when instructed, sliding the phone down to the mid-tibia region. The App then calculates and states the calculated ROM. Patients were then immediately brought to the in-office PT for readings with a manual goniometer. Only one therapist was involved in all measurements. The surgeon and the therapist were blinded to all other measurements made, and each measurement was recorded by the research assistant.

### Statistical analysis

All analyses were performed with R software 3. 4. 4 (R Foundation for Statistical Computing, Vienna, Austria.) Descriptive statistics including mean and standard deviation (SD) were calculated for continuous demographic variables and for ROM measures by each assessor.

Pairwise assessments of agreements among surgeon, App, and the PT were performed. Intraclass correlation coefficients (ICCs) were computed based on the measurements recorded by the surgeon, therapist, and App for both flexion and extension. The ICC model (3,1) was chosen for fixed rater and single measurement per rater. ICC values were interpreted as [3] <0.50, 0.50 to 0.75, 0.75 to 0.90, and >0.90 which indicate poor, moderate, good, and excellent correlation, respectively.

At an alpha level of 0.05, a sample size of approximately 40 patients (with two measurements for each patient) would provide the study with greater than 80% power to detect an ICC of 0.80 compared with the null hypothesis of 0.60. Considering 20% rate of missing measurements, we decided on sample size of 50. Power was calculated using the NCSS PASS 2019, v19.0.2 (NCSS, LLC, Kaysville, UT) with the module “Tests for Intraclass Correlation”.

Limits of agreement (LoA) were calculated using the following formula: LoA = mean difference ± 2 (or 1.3) SD of difference. The interpretation of LoA is that the 95% (or 80%) LoA contain the difference between the two rating methods being compared, for 95% (or 80%) of future measurement pairs.

To assess the effect of BMI on the agreement between methods, we compared the ICC of surgeon vs App by stratifying patients according to the five WHO categories for BMI: below 18.5, 18.5-24.9, 25.0-29.9, 35.0-39.9, above 40. The null hypothesis is that ICCs are the same across BMI categories. *P* values were obtained by a randomization procedure. Specifically, the BMI categories were permutated among subjects 1000 times. Within each permutation, the ICCs were calculated within each BMI category, and the differences in ICCs across the BMI categories were recorded. The 1000 differences in ICCs across BMI categories formed the null distribution based on assuming no difference in the ICCs across BMI categories. The *P* values were estimated by the proportion of randomly generated values that exceed or equal to the observed value in terms of the differences in ICCs across BMI categories. Both extension and flexion were analyzed. Mean and SD of the measurements for each BMI category were also calculated.

A total of 53 patients (20 male, 33 female) were included in this study. Patients had measurements performed on both knees if able. Mean age was 58.9 years (±14.3 years). Mean BMI was 30.8 (±5.5) (Table 1, Table 2).

**Table 1:** Demographics.

Demographics (n = 53)	
Gender	
Male	20
Female	33
Age	58.9 (14.3)
Height (cm)	66.5 (3.9)
Weight (lb)	194.4 (40.04)
Body mass index (BMI)	30.8 (5.5)

The values displayed as mean (standard deviation).

## Results

### Stratification of results by BMI

There were 0, 12, 22, 38, and 32 measurements in patients in the BMI ranges below 18.5, 18.5-24.9, 25.0-29.9, 35.0-39.9, and above 40, respectively. In both flexion and extension, the BMI did not affect the agreement between the measurements significantly—all *P* values were well above 0.05, ranging from 0.14 to 0.86. For flexion, BMI did not show an effect on the extent of agreement among three assessors (*P* = .15); for extension, BMI also did not show an effect on the extent of agreement among three assessors (*P* = .73).

### Interclass correlation analysis

Interclass correlations are shown in Table 3. For extension, the ICC between surgeon and App is 0.64 (ie, within the “moderate” range of 0.50 to 0.75 range as defined in Methods), comparable to that between surgeon and therapist (0.66). For flexion, the ICC between surgeon and App is 0.82 (ie, within the “good” range of 0.75 to 0.90), while the ICC between surgeon and therapist is 0.72, in the “moderate” range.

**Table 2:** Summary of range of motion measurements performed by the surgeon, physical therapist, and digital app (in degrees).

Description	Physician	Therapist	Digital
Extension			
N	104	100	106
mean ± SD (median)	2.7 ± 4.9 (2)	−0.5 ± 4.5 (0)	−1.2 ± 6.5 (−1)
min, max	−6, 28	−13, 25	−16, 26
Flexion			
N	104	100	106
mean ± SD (median)	124.9 ± 13.2 (125)	125.2 ± 12.1 (127)	126.2 ± 13.3 (127.5)
min, max	84, 153	92, 150	84, 155

N, number of subjects; SD, standard deviation.

Note: Measurements were not collected for 3 of 53 patients by the therapist, and for 1 of 53 patients by the surgeon.

**Table 3:** Agreement between the surgeon, therapist, and cell phone app and surgeon for extension and flexion using ICC, with 95% confidence intervals.

ROM	Comparison	ICC	95% CI lower	95% CI upper
Extension	Surgeon vs therapist	0.66	0.57	0.75
Extension	Surgeon vs app	0.64	0.53	0.72
Extension	Therapist vs app	0.58	0.46	0.67
Flexion	Surgeon vs therapist	0.72	0.64	0.79
Flexion	Surgeon vs app	0.82	0.77	0.87
Flexion	Therapist vs app	0.78	0.71	0.84

[3] ICC values <0.50, 0.50-0.75, 0.75-0.90, and >0.90 indicate poor, moderate, good, and excellent correlation, respectively. The correlation between the different methods is moderate overall for extension, and good for flexion.

### Limits of agreement analysis

The LoA are shown in Table 4. For extension, the surgeon tends to give values about 3 degrees greater than therapist and App. The difference between surgeon and therapist can be up to 10 degrees, and the difference between surgeon and App can be up to 13 degrees. For flexion, the bias (mean difference) is small for both comparisons, but the differences can be up to 19 degrees for surgeon vs therapist, and up to 17 degrees between surgeon and App.

**Table 4:** Limits of agreement between the surgeon, therapist, and app, for flexion and extension.

Movement	Comparison	Mean difference^a^	SD difference^a^	95% LOA lower	95% LOA upper	80% LOA lower	80% LOA upper
Extension	Surgeon vs PT	−3.14	3.84	−10.67	4.38	−8.06	1.77
Extension	Surgeon vs app	−3.93	4.92	−13.57	5.70	−10.22	2.36
Extension	PT vs app	−0.64	5.18	−10.79	9.51	−7.27	5.99
Flexion	Surgeon vs PT	0.63	9.37	−17.73	18.99	−11.36	12.62
Flexion	Surgeon vs app	1.29	7.81	−14.02	16.59	−8.71	11.28
Flexion	PT vs app	1.12	8.46	−15.47	17.71	−9.71	11.95

LOA, limit of agreement; SD, standard deviation.

Eighty percent of the differences between surgeon and app are within about 10 degrees for extension and 11 degrees for flexion.

a The difference is the assessment given by therapist minus the assessment given by the surgeon or the assessment given by the app minus the assessment given by the surgeon (or therapist).

### Interpretation of results

When measuring flexion, the correlation between either the surgeon or the therapist with the App was good, whereas the comparison between the surgeon and therapist was moderate. All extension measurement comparisons, between the App, surgeon, and therapist, showed moderate correlation. LoAs showed that 80% of the difference between surgeon and App are within 10 degrees for extension and 11 degrees for flexion. Indeed, ICC for extension between surgeon and App is 0.64 (95% confidence interval [CI]: 0.53-0.74), comparable to 0.66 (95% CI: 0.57-0.5) between surgeon and PT. ICC for flexion between surgeon and App is 0.82 (95% CI: 0.77-0.87), comparable to 0.72 (95% CI: 0.64-0.79) between surgeon and PT (Table 3). The 80% LoA between surgeon and App is −10 to 2 degrees for extension and −9 to 11 degrees for flexion. This is comparable to −8 to 2 degrees for extension and −11 to 13 degrees for flexion between surgeon and PT. Thus, both the agreement and the measurement error between surgeon and App are similar to those between surgeon and PT.

## Discussion

Postoperative ROM is an important measurement after knee surgery. The process of monitoring appropriate postoperative ROM is critical for ensuring progression in the postoperative period. It has been proposed that patients need about 70 degrees of knee flexion to ascend stairs and stand from a seated position and up to 90 degrees to descend stairs [12,13]. Decisions regarding how to manage variations in postoperative ROM are often timely, and at present, the majority of those measurements are made in the surgeon’s office. With the advent of newer technology such as the app used in this study, it may be possible to have more continuous measurements of motion remotely to facilitate timely assessment of deviation. As the data are captured by cell phone, the physician can choose to have them transmitted immediately for review, with alerts if the data are outside a defined standard.

With uncertain times ahead in the midst of the COVID-19 pandemic, digital applications that are able to remotely provide the surgeon and patient with valuable information on their postoperative course will be paramount in total joint arthroplasty. Surgeons around the world are rapidly incorporating telemedicine and remote monitoring into their practice, while attempting to maintain adequate care for their patients. It is unlikely that these newer avenues of patient care will ever be able to replace in-person office visits, but it may become particularly important while we anxiously await a return to the norm.

Manual goniometers have been shown to be accurate in the hands of orthopedic surgeons and therapists [3,6,7,[14], [15], [16]]. Newer technologies, such as accelerometers, are widely available in most modern cell phones and newer wearable technology. These provide a valuable new tool for surgeons and patients alike. The ability of patients to monitor ROM from home at multiple continuous time-points could become a cost-effective and convenient method of monitoring patients in the postoperative period. Furthermore, rather than the traditional office-based monitoring at set postoperative intervals, this technology can provide real time tracking of progression or digression in motion, something previously unavailable, which may not only provide better insight into postoperative motion but also give surgeons a better understanding of certain complications such as arthrofibrosis. The measurements by the surgeon are considered the reference in this study. For postoperative monitoring, measurements are usually made by PTs in clinical visits. An intended use of the app is for patients to take measures themselves at home for postoperative monitoring. Our hypothesis is that the agreement between surgeon and App is similar to the agreement between surgeon and PT.

In order to account for patient body habitus and its potential effect on measurement, we performed an analysis to determine if BMI affected the measurements between the surgeon and digital App. After investigation, we were unable to find a significant effect on measurements when using a BMI cutoff of 35 as differences did not reach statistical significance. Most surgeons would likely admit that these differences are minimal when referring to ROM measurements in the office setting. If a digital App can reliably produce measurements within this range, it may be very beneficial for monitoring patients remotely.

While there is a scant amount of research in this area of study, we were able to compare to a couple of previous studies regarding digital goniometers. In a study by Ferriero et al. [8], a picture of a limb was taken with a cell phone, and an application allowed the clinician to measure the angle based on this photo. It was determined that this method was reliable when compared to a universal manual goniometer. One advantage of the application in our study is that the angle is automatically calculated by the phone App rather than introducing an additional level of variability in the measurement technique described in the aforementioned article. We believe that this attribute makes the smartphone App described here more beneficial for patients to use remotely. In the study by Ferriero et al., there were 35 knees measured. We had a reasonable study size based on the previous literature [8,11]. As the interest in digital applications and remote monitoring continues to grow, more research is needed to further validate this technology.

There are several limitations to this study. First, because the patients are being measured at three different times during the visit, there could be variation in effort, especially in a painful knee which could affect the consecutive measurements. This would underestimate the ICCs and make the LoA wider than concurrent measurement. The design used in the study reflects more pragmatically the intended use scenario. While the technique was the same between the surgeon and the therapist and they were both blinded to the other measurements, the patient was only measured at one time point, and therefore, there may be intraobserver variation that we cannot account for. Manual goniometers are placed on the lateral thigh and calf, while the App data are collected by placing the cell phone on the anterior thigh and sliding it down to the anterior tibia. BMI was meant to be a surrogate for body habitus but may not be the appropriate surrogate and perhaps additional study on thigh circumference would warrant further investigation. We had a reasonable study size based on previous literature [8,11].

## Conclusions

Newer technology that allows for remote patient monitoring continues to provide appealing applications for surgeons and patients. We demonstrated that the studied digital application measuring remote knee ROM was comparable to manual measurements made by either a surgeon and/or PT. Further investigation is necessary to track long-term outcomes and appropriate utilization of this technology in a clinical setting.

## Supplementary data

Conflict of Interest Statement for Shi

Conflict of Interest Statement for Mabee

Conflict of Interest Statement for Frisch

Conflict of Interest Statement for Knapp

Conflict of Interest Statement for Keller

Conflict of Interest Statement for Pillai
