# Supplementary material for: Comparison of a Smartphone App to Manual Knee Range of Motion Measurements
Source: Arthroplast Today. 2022 Apr 4;15:43–6. doi: 10.1016/j.artd.2021.09.005 (PMC8987335; doi:10.1016/j.artd.2021.09.005)
Supplement: Conflict of Interest Statement for Frisch [file mmc3.doc]

# CONFLICT OF INTEREST STATEMENT

***The Journal of Arthroplasty***

(Adopted from the American Academy of Orthopaedic Surgeons disclosure statement)

The following form **must be filled out completely and submitted by each author (example, 6 authors, 6 forms). If no discloser is required, please write/type “none” at the end of each sentence.**

Manuscript Title:

1. Royalties from a company or supplier (The following conflicts were disclosed)

None

2. Speakers bureau/paid presentations for a company or supplier (The following conflicts were disclosed)

Zimmer Biomet

3A. Paid employee for a company or supplier (The following conflicts were disclosed)

None

3B. Paid consultant for a company or supplier (The following conflicts were disclosed)

Zimmer Biomet

3C. Unpaid consultants for a company or supplier (The following conflicts were disclosed)

None

4. Stock or stock options in a company or supplier (The following conflicts were disclosed)

PeerWell, Advanced Orthopaedic Specialties, Smithfield Surgical Partners

5. Research support from a company or supplier as a Principal Investigator (The following conflicts were disclosed)

Zimmer Biomet, DonJoy

6. Other financial or material support from a company or supplier (The following conflicts were disclosed)

None

7. Royalties, financial or material support from publishers (The following conflicts were disclosed)

None

8. Medical/Orthopaedic publications editorial/governing board (The following conflicts were disclosed)

None

9. Board member/committee appointments for a society (The following conflicts were disclosed)

AAHKS YAG Committee

**Each author must sign AND print or type his/her name, date and submit a separate form**

In addition, one BLINDED Conflict of Interest form (no author names used) should be submitted per manuscript with all author disclosures.

Nicholas B Frisch, MD Nicholas B Frisch 2/2/21

Author Name (Print or Type) Author Signature Date
